# Non-participation in a targeted prevention program aimed at lifestyle-related diseases: a questionnaire-based assessment of patient-reported reasons

**DOI:** 10.1186/s12889-022-13382-8

**Published:** 2022-05-13

**Authors:** Christian Leick, Lars Bruun Larsen, Anders Larrabee Sonderlund, Nanna Herning Svensson, Jens Sondergaard, Trine Thilsing

**Affiliations:** 1 Department of Public Health, Research Unit of General Practice, University of Southern Denmark, J.B. Winsløws Vej 9A, 5000 Odense C, Denmark; 2 Steno Diabetes Center Sjælland, Holbæk, Denmark

**Keywords:** Non-participation, Non-response, Health check intervention, Preventive program, Lifestyle, Lifestyle-related diseases, Risk perception, Disease prevention, Primary care

## Abstract

**Background:**

Having an unhealthy lifestyle is associated with a higher risk of developing lifestyle-related diseases. Current evidence suggests that interventions targeting health-risk behaviors can help people improve their lifestyles and prevent lifestyle-related diseases. However, preventive programs are often challenged by low participation rates. Reasons for non-participation include lack of time and/or interest, and/or no perceived need for lifestyle intervention. This study explores causes for non-participation in a sample of people who chose not to take up a targeted preventive program (TOF pilot2 study). Patient-reported reasons as well as sociodemographic characteristics and lifestyle factors are in focus.

**Methods:**

A total of 4633 patients from four Danish GP clinics received an invitation to take part in the TOF pilot2 study. Patients who chose not to participate in the TOF pilot2 study were asked to fill in a questionnaire concerning reasons for non-participation, lifestyle, BMI and self-rated health. Descriptive analyses were used to summarize the results.

**Results:**

A total of 2462 patients (53.1%) chose not to participate in the TOF pilot2 study. Among these, 84 (3.4%) answered the full questionnaire on reasons for not participating, lifestyle, BMI and self-rated health. The most common reasons for non-participation were lack of time, having an already healthy lifestyle, and feeling healthy. Based on their self-reported lifestyle 45 (53.6%) of the non-participants had one or more health-risk behaviors including smoking, unhealthy diet, BMI ≥ 35 and/or sedentary lifestyle and were therefore eligible to receive the targeted intervention at the GP or the MHC in the original TOF pilot2 study.

**Conclusion:**

When planning future preventive programs it is important to know the main reasons for patients to not participate. This study provides rare insight into why people opt out of health interventions and advances the evidence base in this area. Our results may inform efforts to better involve these patients in preventive health programs.

**Trial registration:**

Trial registration number: NCT02797392.

**Supplementary Information:**

The online version contains supplementary material available at 10.1186/s12889-022-13382-8.

## Background

Health-risk behaviors such as smoking, alcohol consumption, sedentary behavior, and unhealthy diet are associated with increased risk of a range of lifestyle-related diseases, including type 2 diabetes (T2DM), cardiovascular disease (CVD), and chronic obstructive pulmonary disease (COPD) [1–4]. These diseases have high mortality rates and are especially prevalent in upper middle- and high-income countries [5]. Studies suggest that preventive lifestyle programs that target the at-risk population can help mitigate the development of lifestyle-related diseases [6, 7]. In Denmark primary disease prevention falls under the responsibility of the municipalities. General practitioners (GP) have a key role in preventive programs against lifestyle-related diseases as they are generally familiar with their patients and their social circumstances [8]. Nonetheless, preventive programs are often hampered by low participation rates and disproportionately high uptake among women, the elderly, as well as people with better health and higher socioeconomic status [9]. At a population level, this signifies important shortcomings in intervention design and reach. It also highlights the need for insight into why people who might benefit from preventive health programs decline invitations to participate [10].

Information about non-participating populations is important for the design of targeted and tailored interventions. However, only few studies report information about non-participants and their reasons for not participating [9, 11]. This study focuses on patient-reported reasons for non-participation, sociodemographic characteristics and self-reported lifestyle among non-participants in a targeted preventive health check intervention (TOF pilot2).

## Methods

### Setting and study design

The TOF pilot study 2 (TOF is a Danish acronym for *Early Detection and Prevention*) was conducted in the Region of Southern Denmark in collaboration with four GP clinics and two municipalities, Middelfart and Haderslev. In Denmark, primary disease prevention falls under municipal purview. Municipalities thus offer courses in smoking cessation and physical activity, dietary advice and treatment of alcohol addiction. Secondary disease prevention, such as preventive medical treatment and chronic disease management, is mainly undertaken by GPs. This study was conducted as a non-randomized feasibility study and included all eligible patients from the four GP clinics.

The intervention comprised a general intervention offered to all participants and a targeted intervention for those participants at high risk of lifestyle-related disease. As part of the general intervention, a personal digital health profile was created for each participant. The digital health profile was based on participant survey responses and information from the electronic patient records at the GP and offered information about risk of disease and advice for lifestyle changes. In addition to the health profile, the targeted intervention included a health assessment at the GP for high-risk patients and follow-up with treatment intervention at the GP, tailored health services at a municipal health center (MHC), or both. Or the offer of tailored health services at a MHC for patients with health-risk behavior [12, 13].

In October 2018, a total of 4633 patients from the four GP clinics received an invitation to take part in the study. Patients were eligible to participate if they were born between 1959–1988, lived in the municipalities of Haderslev or Middelfart, had a digital mailbox, and had not been invited to the first TOF pilot study (TOF pilot1). The invitation was sent on behalf of the GP and the MHC to patients’ digital mailboxes (e-Boks). A digital mailbox is a public Danish system for secure electronic communication between residents in Denmark and public authorities and other trusted organizations (e.g., banks) [14].

The invitation contained a link to a password-protected personal website with information about the study and a consent form. Non-response triggered up to two reminders with two interval. The initial invitation as well as the reminders contained a link to a questionnaire for those patients who chose not to take part in the study (non-participation questionnaire). Participation was voluntary and patients were informed that non-participation had no influence on their treatment options.

### Development of the non-participation questionnaire

#### Question on reasons for non-participation

Initially, 17 reasons for non-participation were formulated by the research group. The items were based on current evidence about patient-reported factors for attendance and non-attendance at preventive health checks [15–18]. The items covered project-related as well as personal and practical factors.

#### Patient and public involvement

The questions were tested for comprehensibility, relevance, and coverage by target group representatives. Men and people with low educational level were purposely sampled as these subgroups of the general population are particularly likely to decline preventive interventions [9, 11, 13, 19]. The recruitment strategy centered on Facebook and Instagram ads. In addition, attendees at a Meeting Place for Men (a social connectedness initiative for men) in the town of Sønderborg were contacted directly. Ultimately, 10 citizens aged 34–57 (three women, seven men) were recruited. Eight participants had no formal education beyond elementary school, one was a primary school teacher, and one was a printmaker [13].

An invitation to the TOF pilot study 2 and the questionnaire draft were sent by mail to the target group representatives. Group members were instructed to imagine that they received the invitation to participate in TOF Pilot2 study and decided not to participate. They were then instructed to access the non-response questionnaire by clicking a link in the invitation, “*If you do not want to participate, please click here and let us know why*”. Group members were asked to indicate if any of the questions overlapped and invited to suggest inclusion of additional questions. The questions were adjusted accordingly, and one option on reasons for non-participation were added which was “*I am already in treatment for a lifestyle-related disease”.*

Beyond reasons for non-participation, the questionnaire contained items on lifestyle, BMI and self-rated health. Questions on physical activity were adapted from the Danish Diabetes Risk model [20]. Questions on smoking status were derived from the COPD-PS screener [21] and the Heartscore BMI score [22]. Diet was assessed with the Swedish National Guidelines on Disease Prevention [23], and alcohol consumption was gauged according to official Danish recommendations for low-risk alcohol consumption [24].

Hence the final questionnaire contained 11 questions all of which made use of non-forced answers. (for resulting questionnaire see additional file 1).

#### Analysis

Information on health-risk behavior (smoking status, alcohol consumption, physical activity, diet and BMI) were dichotomized. Smoking status was defined in terms of current smoking status (yes/no). ‘Current smoker’ included daily and occasional smokers while ‘non-smoker’ referred to people who had quit smoking or never smoked [12]. Alcohol consumption was divided into low-risk or high-risk alcohol consumption (> 14 and 21 units/week for women and men, respectively [24]). Level of physical activity was dichotomized into ‘sedentary’ and ‘physically active activities during leisure time’. Activity during leisure time was defined as four hours or more of low- to high-intensity leisure-time activity (gardening, walking the dog, cycling, etc.) a week. Sedentary leisure time was defined as reading, watching television or other sedentary activities. Diet was dichotomized as unhealthy diet or otherwise. Unhealthy diet was measured as a score of four or lower on a 12-point dietary scale [23]. The respondents were asked to type in their height and weight. Respondent BMI was then calculated and categorized as either BMI < 35 or BMI ≥ 35. Self-rated health was assessed by the following question “*In general, would you say your health is*”: Excellent (1), Very good (2), Good (3), Fair (4) or Poor (5). This was dichotomized into “Excellent, Very good or Good self-rated health” and “Fair or Poor self-rated health”.

#### Register information

Patient demographic information on sex, age, country of origin, educational level, occupational status, and family income was retrieved from the Danish National Bureau of Statistics (Statistics Denmark) for the entire study population and linked at the individual level.

Age was determined at the time of invitation and categorized in 10-year age brackets (29–39, 40–49, 50–60). Country of origin was retrieved for the year 2018 and categorized as Danish, Western, or Non-western origin. Highest attained educational level was retrieved for October 2018 and categorized into five groups: Secondary school, High school, Vocational education, Higher education, and Higher education – master’s level. Educational level was then dichotomized into ‘highest educational level: “secondary school”, or “High school, vocational education, higher education and higher education – master level”. Employment status was retrieved for November 2018 and categorized according to the equivalence scales into five categories: Employed, Self-employed, Unemployed/on benefits, social welfare recipients, and other. The category “other” represents, for instance, unemployed persons from a family that relies on one income only [13]. Employment status was dichotomized as “unemployed/on benefits, social welfare recipients or other”, and “employed or self-employed”. Family income was retrieved for 2013–2018, defined by the mean annual net income of the household, and categorized into quartiles. Subsequently, family income was dichotomized (Low income: 1 = lowest quartile, 0 = above lowest quartile) [12, 13].

#### Statistical analyses

Descriptive analysis was used to summarize the patient’s demographic characteristics, their reasons for non-participation, smoking, alcohol consumption, diet, physical activity, BMI and their self-rated health. To examine the differences in demographic characteristics between non-participants who answered the non-participation questionnaire and those who did not, univariate and multivariate logistic regression analyses were performed. In the multivariate logistic regression analyses the demographic characteristics were adjusted for gender and age. Missing values were excluded in the analyses. Odds Ratios (crude and adjusted), p-values with a 0.05 significance level and 95% confidence intervals were reported. Stata version 16.0 was used for the analyses.

## Results

**Fig. 1:**
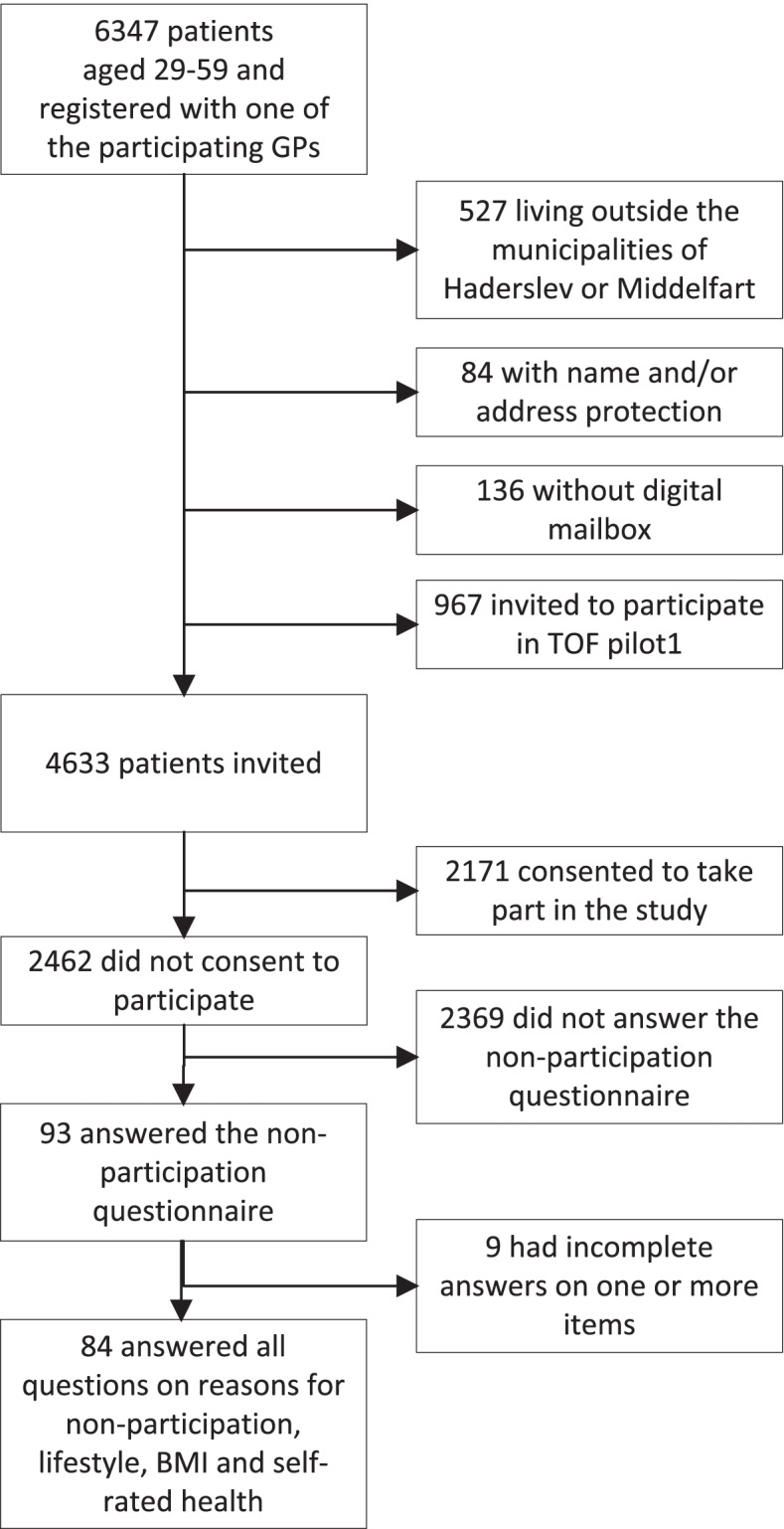
Flowchart of the TOF pilot2 study population

Of the 4633 patients who were invited to the TOF pilot2 study, 2462 (53.1%) chose not to participate [13]. Of these, 93 (3.8%) answered some or all of the questions in the non-participation questionnaire. Eighty-four (3.4%) (men = 37 and women = 47) answered the questions on reasons for non-participation as well as questions on lifestyle, BMI and self-rated health (Fig. 1).

### Characteristics of non-participants compared to participants

The proportion of men was higher among non-participants than participants. Further non-participants were younger, more likely to be of Western or Non-Western origin, and more likely to have secondary school as their highest educational attainment. In terms of SES, non-participants were more likely than participants to be in the lowest income quartile and more likely to be unemployed/on benefits/social welfare recipients than their participating counterparts (additional file 2).

### Characteristics of responders and non-responders to the non-participation questionnaire

Table 1 shows the demographic characteristics of non-participants who answered the non-participation questionnaire (responders) and non-participants who did not (non-responders). Responders were older and more likely to be women but did not differ significantly from non-responders with regard to country of origin, level of education, occupational status, or family income (Table 1).

**Table 1:** Demographic characteristics of responders and non-responders to the non-participation questionnaire

	Respon-ders	Non-respon-ders	Total number of non-partici-pants	Mis-sing, n (%)	Logistic regression					
					Crude			Adjusted^b^		
n (%)	93 (3.8)	2369 (96.2)	2462 (100)		OR	95% Cl	*p*	OR	95% Cl	*p*
**10-year age groups, n (%)**				0 (0.0)						
29–39 years	17 (18.3)	804 (33.9)	821 (33.4)		1					
40–49 years	34 (36.6)	892 (37.7)	926 (37.6)		1.80	1.00–3.25	0.05			
50–60 years	42 (45.2)	673 (28.4)	715 (29.0)		2.95	1.66–5.23	< .001			
**Gender, n (%)**				0 (0.0)						
Female	58 (58.1)	1112 (46.9)	1166 (47.4)		1					
Male	39 (41.9)	1257 (53.1)	1296 (52.6)		0.64	0.42–0.97	0.036			
**Country of origin, n (%)**				15 (0.6)						
Denmark	84 (90.3)	2050 (87.1)	2134 (87.2)		1			1		
Western	< 5	99 (4.2)	101 (4.1)		0.49	0.12–2.03	0.33	0.55	0.13–2.26	0.40
Non-western	7 (7.5)	205 (8.7)	212 (8.7)		0.83	0.38–1.83	0.65	0.92	0.42–2.03	0.84
**Highest educational attainment, n (%)**				106 (4.3)						
Highschool, vocational education, higher education, higher education—master level	73 (82.0)	1799 (79.4)	1872 (79.5)		1			1		
Secondary school	16 (18.0)	468 (20.6)	484 (20.5)		0.84	0.49–1.46	0.54	0.83	0.48–1.45	0.52
**Employment status, n (%)**				13 (0.5)						
Unemployed/ on benefits, social welfare recipients or other^a^	27 (29.0)	508 (21.6)	535 (21.9)		1			1		
Employed, self-employed	66 (71.0)	1848 (78.4)	1914 (78.2)		0.67	0.42–1.06	0.09	0.70	0.44–1.11	0.13
**Family income, n (%)**				15 (0.6)						
> Lowest quartile	72 (77.4)	1633 (69.3)	1705 (69.7)		1			1		
Lowest quartile	21 (22.6)	721 (30.6)	742 (30.3)		0.66	0.40–1.08	0.10	0.75	0.45–1.24	0.26

^a^ “Other” covers e.g., unemployed persons from a family that relies on one income only

^b^Adjusted for age and gender

### Reasons for non-participation

#### Reported reasons for non-participation

The reported reasons for not participating in the TOF pilot study 2 are shown in Table 2 and divided into the following overarching categories (bold in the table): Time constraints; No perceived need for a health check; Unclarity of the program; Privacy concerns; Do not want to know results/fear of bad results; Do not want to participate because of the program/parts of the program; and Already in treatment. The most common specific reasons for not participating were lack of time (*n* = 25 (29.8%)), having a healthy lifestyle (*n* = 24 (28.6%)), and feeling healthy (*n* = 20 (23.8%)). For men, the most frequently reported reason was lack of time (*n* = 13 (35.1%)), whereas for women it was having a healthy lifestyle (*n* = 17 (36.2%)). Only very few (< 5) respondents cited fear of a negative health check result as a reason for not participating. No patients reported that they were either too young or too old to benefit from a health check.

**Table 2:** Patient reasons for not participating grouped into broader categories

I do not want to participate in the study because … (n(%))	Men (*n* = 37)	Women (*n* = 47)	Total (*n* = 84)
**Time constraints**	**13 (35.1)**	**12 (25.5)**	**25 (29.8)**
I do not have time to participate	13 (35.1)	12 (25.5)	25 (29.8)
**No perceived need for a health check**	**18 (48.7)**	**28 (59.6)**	**46(54.8)**
I already have a healthy lifestyle	7 (18.9)	17 (36.2)	24 (28.6)
I feel healthy	10 (27)	10 (21.3)	20 (23.8)
I get regularly health checks at my GP	7 (18.9)	9 (19.2)	16 (19.1)
I can change my lifestyle if I feel the need for it	6 (16.2)	9 (19.2)	15 (17.9)
I think I am too young to get something out of a health check	0 (0)	0 (0)	0 (0)
I think I am too old to get something out of a health check	0 (0)	0 (0)	0 (0)
**Unclarity of the program**	**8 (21.6)**	**9 (19.2)**	**17 (20.2)**
It is unclear for me what the intervention is about	8 (21.6)	9 (19.2)	17 (20.2)
**Privacy concerns**	**10 (27)**	**8 (17)**	**18 (21.4)**
I do not wish that information from my journal at my own GP will be given to this intervention	5 (13.5)	7 (14.9)	12 (14.3)
I am afraid others will get access to the results e.g. insurance companies	NS	NS	9 (10.7)
**Do not want to know results/fear of bad results**	**10 (27)**	**10 (21.3)**	**20 (23.8)**
Participation in this intervention will make me unnecessarily nervous	5 (13.5)	6 (12.8)	11 (13.1)
I do not want to know my risk of developing a lifestyle-related disease	6 (16.2)	5 (10.6)	11 (13.1)
I am afraid the results of a health check will be negative	NS	NS	< 5
**Do not want to participate because of the program/parts of the program**	**9 (24.3)**	**13 (27.7)**	**22 (26.2)**
I do not believe the intervention can help me get a healthier lifestyle	NS	NS	10 (11.9)
I do not like when others interfere with my lifestyle	NS	NS	9 (10.7)
I do not want to change my lifestyle	NS	NS	7 (8.3)
I do not want to get medicine if that is what my GP recommends	NS	NS	< 5
**Already in treatment**	NS	NS	8 (9.5)
I am already in treatment for a lifestyle-related disease	NS	NS	8 (9.5)

*NS* Not shown due to low number in cell

The respondents were also given the opportunity to report their own reason for not participating Twenty-eight patients used this option and the reasons reported were grouped into broader categories: “Already diagnosed with a chronic disease” (*n* = 5), “Does not want to participate” (*n* = 5), “Does not have the energy to participate (*n* = 4), Receives regular health checks at GP” (*n* = 3), “Technical difficulties with questionnaire (*n* = 2), Geographical reasons (*n* = 2), “Does not perceive themselves as in need for a health check” (*n* = 2), “Not satisfied with GP´s in general” (*n* = 2), “Fear of being stigmatized” (*n* = 1), “Cannot participate because of poor mobility” (*n* = 1) and “Privacy concerns” (*n* = 1).

#### Perceived need for health check, health behavior, and BMI

Of the responders who answered the questions on lifestyle (*n* = 91), 21 (23.1%) were current smokers, 14 (15.4%) had an unhealthy diet, 17 (18.7%) had a sedentary lifestyle, and none had high-risk alcohol intake. Among the participants who answered the questions on BMI and self-rated health (*n* = 89), 10 (11.2%) had a BMI ≥ 35 and 16 (18%) had fair or poor self-rated health.

Table 3 shows the proportion of patients with health-risk behaviors and fair or poor self-rated health among the non-participant respondents with no perceived need for a health check (*n* = 46) compared to respondents reporting other reasons for non-participation (*n* = 38)(See Table 2). We include this information to provide a sense of those responders who chose not to participate because they perceived no need to do so, but who nonetheless represent the target group for the intervention by virtue of their health-risk behaviors.

**Table 3:** Health-risk behavior, BMI, and self-rated health among non-participant respondents

	No perceived need for a health check (n (%))	Other reasons for non-participation (n (%))	Total (n (%))	Missing (n (%))
	46 (54.8)	38 (45.2)	84 (100)	
Current smoker, n (%)	10 (21.7)	10 (26.3)	20 (23.8)	2 (2.4)
Unhealthy diet, n (%)	5 (10.9)	8 (21.1)	13 (15.5)	2 (2.4)
Sedentary lifestyle, n (%)	6 (13)	8 (21.1)	14 (16.7)	2 (2.4)
High-risk alcohol intake, n (%)	0 (0)	0 (0)	0 (0)	2 (2.4)
BMI (kg/m^2^) ≥ 35, (%)	2 (4.4)	7 (18.4)	9 (10.7)	3 (3.6)
Fair or poor self-rated health, n (%)	4 (8.7)	7 (18.4)	11 (13.1)	3 (3.6)
Number of patients with one or more health risk behaviors^a^, n (%)	21 (45.7)	24 (63.2)	45 (53.6)	

^a^Some patients had more than one health risk behavior e.g. both a current smoker and maintained a sedentary lifestyle

The number of patients with one or more health risk behaviors were slightly lower among patients with no perceived need for at health check (45.7%), than among patients reporting other reasons for non-participation (63.2%). In total, 45 (53.6%) of the respondents had one or more health-risk behaviors and were therefore eligible to receive the targeted intervention at the GP or the MHC.

## Discussion

The aim of this study was to examine patient-reported reasons for non-participation as well as sociodemographic characteristics and self-reported lifestyle among non-participants in the TOF pilot 2 study.

Of the patients invited to take part in the study, about half declined. Consistent with many other studies focusing on health-check interventions [9, 11, 19, 25–27], non-participants differed from the participants by being disproportionately male, younger, and with lower SES.

Responders (i.e., non-participants who answered the questions on reasons for non-participation) were comparable to non-participants on all sociodemographic characteristics except gender and age. The main findings of the study are discussed below.

### Time constraints

The most common reason cited for non-participation was a lack of time (29.8%). This resonates with previous findings [15, 16, 18]. People in the work force have previously reported lack of time because of a busy work schedule as a barrier to exercise [28] and maintaining a healthy diet [29]. Therefore, as the target group of the present study comprised working-age patients (29–60 years), time constraints related to a busy work schedule most likely also influenced the likelihood of intervention uptake. A possible solution to reach people in the work force could be to implement preventive programs at workplaces. Indeed, recent workplace interventions have shown promising results in terms of physical activity, dietary behavior, healthy weight [30], smoking cessation [31], and cardiovascular-disease-risk reduction [32].

A notable finding from our study concerns the fact that almost 30% of non-participants were unemployed/on benefits. The existing literature concerning time constraints as a reason for non-participation has not included a specific focus on the unemployed. But a study that investigated reasons for non-participation in a health promotion program targeting low-income households, including unemployed, reports circumstances such as family illness as a barrier for participation [33].

### Mismatch between perceived and actual risk

The reasons “*I already have a healthy lifestyle*” and “*I feel healthy*” are the second and third most frequent reasons provided by non-participants. Indeed, 40.5% of the patients reported one or both, which makes this the most cited reason for non-participation. Consistent with these results, other studies have reported the feeling of healthiness as a factor in non-participation [9, 18]. To have a healthy lifestyle or to feel healthy is a subjective assessment and it is possible that their risk perception of developing a lifestyle-related disease is inaccurate. Previous studies have shown a mismatch between actual risk and risk perception of developing lifestyle-related diseases where participants’ perceived risk was lower than the actual risk [8, 34, 35]. The results of present study indicate that 21 (45.7%) non-participants perceived no need for a health check, but nonetheless had one or more health-risk behaviors. In the TOF pilot study 2 these patients would have received an offer of a targeted intervention at the GP or the MHC. Though it is challenging to reach out to patients who already feel healthy, even though they show health-risk behavior, it is important as such patients might not engage in the necessary lifestyle changes. A possible solution could be for health professionals to educate their patients about actual and perceived risk of health behaviors [36]. It may be challenging to get patients who believe that they are healthy to seek out and engage in such education. However, previous studies have indicated that completing an online risk calculator with a health care professional, who can help interpret the results in the context of patient preferences, needs and expectations, is preferable among patients [36–38].

### Unclarity of the program

Confusion about the aim and content of the intervention was reported by 20.2% of the non-participants. This aligns with another study in which lengthy and ambiguous information about the intervention represented a key barrier for uptake and participation [39]. That study recommended shortening participant information as much as possible, but recognized that this might be difficult as ethics committees generally require specific and wordy details to be incorporated into participant information [39].

### Privacy concerns

The reasons “*I do not want my medical records to be shared with the research team*” and “*I am concerned that others will get access to the results e.g., insurance companies*” were reported as reasons for non-participation by 14.3% and 10.7%, respectively. Patient medical records are generally perceived as more private than other types of personal information, and patients may therefore be less likely to disclose this information [40, 41]. Patients may worry their information could end up in the wrong hands, such as insurance companies [41, 42]. One study indicated that participants were willing to disclose health information in their physicians office [41] and even though it could also be a possibility in this program it would be a lot more time consuming and resource demanding compared to the current set up.

### Do not want to know results/Fear of bad results

Both of the reasons “*Participation in this intervention will make me unnecessarily nervous*” and “*I do not want to know my risk of developing a lifestyle disease”* were reported as reasons for non-participation by 13.1% of respondents. Similar results are evident in other studies regarding attendance at GP health checks [43–45]. More direct and clear communication from the GP concerning risk behavior and the importance of health checks may mitigate this level of willful ignorance and galvanize the patient into preventive action. Indeed, previous studies show that non-responders seems to value a personal approach about their own risk perception from the GP [46].

### Do not want to participate because of the program/parts of the program

Reasons as “*I do not believe the intervention can help me get a healthier lifestyle”* and “*I do not like others interfering with my lifestyle”* were reported as reasons for non-participation by 11.9% and 10.7%, respectively. Several factors could underly these reasons for non-participation, including for example, dissatisfaction with the specific program design or operation – an issue seen in a previous study [9]. However, given the fundamental nature of these two reasons for non-participation – that is, disbelief in the efficacy of the intervention, and a more general rejection of outside interference with one’s lifestyle – persuading this subgroup to participate would presumably always be a hard sell. Still, as mentioned earlier a personal approach from the GP should be explored further as a possible solution to increase participation within this group [46].

### Strengths and limitations

A strength of this study is the involvement and response from a group that is usually hard to reach (i.e., non-participants). The reasons for not participating in a preventive program targeting lifestyle-related diseases is a key factor in the implementation of current and future preventive programs.

While the low response rate of 3.8% is a clear limitation to the external validity of the present study, we emphasize the fact that non-responders and responders to the non-participation questionnaire were comparable on most of the measured demographic parameters including country of origin, level of education, occupational status and family income. This suggests that despite the low response rate, responders are at least somewhat representative of the population of interest. Nonetheless, there is a possibility that this study could be subject to selection bias as responders to the non-participation questionnaire may differ systematically from the other non-responders on other factors than demographic ones. For instance, the digital nature of the non-response questionnaire might represent a barrier for people who lack the necessary IT skills and literacy. This was also considered a limiting factor of the TOF pilot1 study [47]. Further, questionnaire respondents may have had a more positive attitude towards participation in the TOF pilot study 2 than other non-participants. This could influence the results concerning reasons for non-participation and maybe over -or underestimate some of the reasons. This factor and the low response rate was also reported in a recent similar study [46]. Furthermore, the questionnaire was self-reported which could cause recall bias and/or information bias as there is a possibility that the patients do not remember or misunderstand some of the questions.

Another limitation relates to the fact that this study did not measure non-participants willingness to participate in future preventive programs against lifestyle-related diseases. Therefore, we cannot know if the reservations described only pertained to the current set up or point in time.

### Implications

Although the GPs were very much involved in the design and planning of the TOF intervention the results in this study could indicate that there is still room for improvement. The existing literature indicate that participation rates may increase if the GP plays a bigger role in the intervention. Both regarding the invitation process as non-responders seems to value a personal approach from the GP but also to establish a dialogue on actual risk and risk perception of health behavior with the patients as there seems to be mismatch [8]. By doing this, patients might be better suited to assess the actual health risks associated with their behavior. In turn, this might encourage them to change their lifestyle – for example by participating in a preventive program.

As the most common reason for non-participation relates to time constraints, a program that could be implemented at workplaces might increase participation rates. Regarding the current set up it should be a possibility for people to receive offers of a targeted intervention at the GP or the MHC out of office hours. Furthermore, given the large proportion of unemployed non-participants, a program targeting the unemployed could be a solution for reaching this group more effectively. In terms of future similar health promotion programs our results, regarding time constraints being the most common reason, suggests that it is important to remove structural barriers and create an environment in which it becomes possible or easier for the individual to participate.

Finally, to better understand the reasons for non-participation, a qualitative study would conceivably provide a wider and richer perspective on the underlying reasons for non-participation. Knowledge about the reasons for non-participation is not exhaustive and warrants further evidence in order to target the population who may benefit from preventive interventions.

## Conclusion

In the present study, the most common reasons for non-participation in a targeted preventive health check intervention were “time-constraints” and “already feels healthy”. However, regarding the reason “already feels healthy”, results on the patients lifestyle indicates that patients at risk for lifestyle diseases do not perceive themselves as being at risk so there might be an incongruence between perceived risk and actual risk.

Our results extends existing knowledge on reasons for non-participation and highlights future areas of research.

## Supplementary Information

**Additional file 1. **Questionnaire.

**Additional file 2. **Characteristics of non-participants compared to participants.

## Data Availability

The data that support the findings of this study are not publicly available. Data will however be available from the authors upon reasonable request and with permission from the Danish Data Protection Agency. If data from this study is requested please contact Christian Leick.

## Abbreviations

BMI: Body mass Index
COPD: Chronic obstructive pulmonary disease
CVD: Cardiovascular disease
GP: General practitioner
MHC: Municipal health center
T2DM: Type 2 diabetes
TOF: TOF is a Danish acronym for Early Detection and Prevention (in Danish: Tidlig Opsporing og Forebyggelse)
