# Supplementary material for: Non-participation in a targeted prevention program aimed at lifestyle-related diseases: a questionnaire-based assessment of patient-reported reasons
Source: BMC Public Health. 2022 May 13;22:970. doi: 10.1186/s12889-022-13382-8 (PMC9107116; doi:10.1186/s12889-022-13382-8)
Supplement: Supplementary file 1 — Additional file 1. Questionnaire. [file 12889_2022_13382_MOESM1_ESM.docx]

## Additional file 1: Questionnaire

*This study should give us knowledge about what may be the reason one does not want to participate in the TOF pilot2 study. It takes approximately 5 min. to answer the questionnaire. All answers are treated confidentially. It is completely voluntary to participate. Please note that by answering this questionnaire you have not been deregistered from the project. If you want to unsubscribe from the project, click on the link at the bottom of the invitation you received in your e-Box.*

**1. Tick the statement(s) that best describe the reason why you do not want to participate in the project. I do not want to participate in the study because…**

- It is unclear for me what the intervention is about
- I do not believe the intervention can help me get a healthier lifestyle
- I already have a healthy lifestyle
- I can change my lifestyle if I feel the need for it
- I do not want to change my lifestyle
- I feel healthy
- I do not want to know my risk of developing a lifestyle-related disease
- I get regular health checks at my GP
- I am afraid the results of a health check will be negative
- I think I am too young to get something out of a health check
- I think I am too old to get something out of a health check
- I do not like when others interfere with my lifestyle
- I am afraid others will get access to the results e.g. insurance companies
- I do not have time to participate
- I do not want information from the electronic patient record at my GP is passed on to this intervention
- Participation in this intervention will make me unnecessarily nervous
- I do not want to get medicine if that is what my GP recommends
- I am already in treatment for a lifestyle-related disease
- If other reasons, please specify

*Here are some questions about you, your health and your lifestyle*

**2. In general, would you say your health is…**

- Excellent
- Very good
- Good
- Fair
- Poor

**3. How tall are you? (cm)**

**4. What is your weight? (kg)**

**5. Do you smoke?**

- Daily smoker
- Occasionally smoker
- Quitted smoking in less than 6 months
- Quitted smoking in more than 6 months
- Never smoked

**6. How many units of alcohol do you drink on average per week in a regular week?**

- 0
- 1-6
- 7-13
- 14-20
- 21 or more

**7. How often do you eat vegetables and/or root vegetables? Fresh as well as frozen.**

- Twice daily
- Daily
- Sometimes during the week
- Once a week or less

**8. How often do you eat fruit and/or berries? Fresh, frozen, canned or juice/smoothie.**

- Twice daily
- Daily
- Sometimes during the week
- Once a week or less

**9. How often do you eat seafood as a main course?**

- Three times a week or more often
- Twice a week
- Once a week
- A couple times a month or less

**10. How often do you eat pastry, sweets, chips and/or drink soda?**

- Daily
- Almost every day
- A couple times a week
- Once a week or less

**11. If you look at the past year, what would you say fits best as a description of your physical activity in your spare time?**

- Trains hard and practices competitive sports regularly several times a week
- Exercises sport or does heavy gardening or similar at least 4 hours a week
- Walks, bikes or does other light exercise at least 4 hours a week
- Reads, watches television or have other sedentary activities
