# Supplementary material for: Non-participation in a targeted prevention program aimed at lifestyle-related diseases: a questionnaire-based assessment of patient-reported reasons
Source: BMC Public Health. 2022 May 13;22:970. doi: 10.1186/s12889-022-13382-8 (PMC9107116; doi:10.1186/s12889-022-13382-8)
Supplement: Supplementary file 2 — Additional file 2. Characteristics of non-participants compared to participants. [file 12889_2022_13382_MOESM2_ESM.docx]

# Additional file 2: Characteristics of non-participants compared to participants

|  | Participants | Non-participants | N | Mis-sing, n (%) | Logistic regression | | | | | | |
| --- | --- | --- | --- | --- | --- | --- | --- | --- | --- | --- | --- |
|  |  |  |  |  | Crude | | | Adjusted** | | | |
| n (%) | 2171  (46.9) | 2462 (53.1) | 4633 (100) |  | OR | 95% Cl | *p* | OR | 95% Cl | | *p* |
| **10-year age groups, n (%)** |  |  |  | 0  (0.0) |  |  |  |  |  |  | |
| 29-39 years | 426 (19.6) | 821 (33.4) | 1247  (26.9) |  | 1 |  |  |  |  |  | |
| 40-49 years | 850 (39.2) | 926 (37.6) | 1776  (38.3) |  | 1.77 | 1.52-2.05 | <.001 |  |  |  | |
| 50-60 years | 895 (41.2) | 715 (29) | 1610  (34.8) |  | 2.41 | 2.07-2.81 | <.001 |  |  |  | |
| **Gender, n (%)** |  |  |  | 0  (0.0) |  |  |  |  |  |  | |
| Male | 903 (41.6) | 1296 (52.6) | 2199 (47.5) |  | 1 |  |  |  |  |  | |
| Female | 1268 (58.4) | 1166 (47.4) | 2434 (52.5) |  | 1.56 | 1.39-1.75 | <.001 |  |  |  | |
| **Country of origin, n (%)** |  |  |  | 21 (0.5) |  |  |  |  |  |  | |
| Denmark | 2056 (95) | 2134 (87.2) | 4190 (90.9) |  | 1 |  |  | 1 |  |  | |
| Western | 56 (2.6) | 101  (4.1) | 157  (3.4) |  | 0.58 | 0.41-0.80 | 0.001 | 0.63 | 0.45-0.89 | 0.008 | |
| Non-western | 53  (2.4) | 212 (8.7) | 265  (5.8) |  | 0.26 | 0.19-0.35 | <.001 | 0.23 | 0.21-0.39 | <.001 | |
| **Highest educational attainment, n (%)** |  |  |  | 155  (3.4) |  |  |  |  |  |  | |
| Secondary school | 274 (12.9) | 484 (20.5) | 758  (16.9) |  | 1 |  |  | 1 |  |  | |
| Highschool, vocational education, higher education, higher education - master level | 1848 (87.1) | 1872 (79.5) | 3720 (83.1) |  | 0.84 | 0.49-1.46 | 0.54 | 0.83 | 0.48-1.45 | 0.52 | |
| **Employment status, n (%)** |  |  |  | 13 (0.5) |  |  |  |  |  |  | |
| Unemployed/ on benefits, social welfare recipients or other* | 308 (14.2) | 535 (21.9) | 843  (18.3) |  | 1 |  |  | 1 |  |  | |
| Employed, self-employed | 1861 (85.8) | 1914 (78.2) | 3775 (81.8) |  | 1.69 | 1.45-1.97 | <.001 | 1.82 | 1.55-2.13 | <.001 | |
| **Family income, n (%)** |  |  |  | 21 (0.5) |  |  |  |  |  |  | |
| Lowest quartile | 352 (16.3) | 742 (30.3) | 1094  (23.7) |  | 1 |  |  | 1 |  |  | |
| > Lowest quartile | 1813 (83.7) | 1705 (69.7) | 3518 (76.3) |  | 2.24 | 1.94-2.59 | <.001 | 2.05 | 1.77-2.38 | <.001 | |

* “Other” covers e.g., unemployed persons from a family that relies on one income only

** Adjusted for age and gender
